# Effect of Laryngeal Mask Airway Insertion on Intraocular Pressure Response: Systematic Review and Meta-Analysis

**DOI:** 10.1155/2020/7858434

**Published:** 2020-07-09

**Authors:** Mohammed Suleiman Obsa, Zewde Zema kanche, Robera Olana Fite, Tilahun Saol Tura, Bulcha Guye Adema, Aseb Arba Kinfe, Melkamu Worku kercho, Kebreab Paulos chanko, Getahun Molla Shanka, Atkuregn Alemayehu Lencha, Gedion Asnake Azeze, Lolemo Kelbiso Hanfore, Nefsu Awoke Adulo, Blen Kassahun Dessu, Getahun Dendir Wolde, Shimelash Bitew Workie

**Affiliations:** ^1^School of Anesthesia, Wolaita Soddo University, Wolaita Soddo, Ethiopia; ^2^School of Pharmacy, Wolaita Soddo University, Wolaita Soddo, Ethiopia; ^3^School of Nursing, Wolaita Soddo University, Wolaita Soddo, Ethiopia; ^4^School of Midwifery, Wolaita Soddo University, Wolaita Soddo, Ethiopia; ^5^School of Medicine, Wolaita Soddo University, Wolaita Soddo, Ethiopia; ^6^School of Public Health, Wolaita Soddo University, Wolaita Soddo, Ethiopia

## Abstract

**Background:**

Use of laryngeal mask airway as an alternative to the endotracheal tube has attracted the attention of several workers with regard to intraocular pressure changes. However, the previous studies have reported different results while comparing intraocular pressure, following insertion of laryngeal mask airway or the endotracheal tube. Therefore, this systematic review and meta-analysis was aimed to generate the best possible evidence on the intraocular pressure response to endotracheal tube intubation and laryngeal mask airway insertion.

**Methods:**

Electronic databases like PubMed, CINAHL, EMBASE, Google Scholar, Cochrane library databases, and Mednar were used. All original peer-reviewed papers which reported the mean and standard deviation of IOP before and after airway instrumentation in both groups were included. Two reviewers independently extracted the data using a standardized data extraction format for eligibility and appraised their quality. Data were analyzed using the STATA version 14 software. The pooled standard mean difference was estimated with the random-effect model. Heterogeneity between studies was assessed by the *I*^2^ statistics test. A subgroup analysis was done to assess the source of variation between the studies.

**Result:**

A total of 47 research papers were reviewed, of which, six studies were finally included in this systematic review and meta-analysis. The overall pooled standard mean difference of intraocular pressure was 1.30 (95% CI, 0.70, 1.90), showing that LMA insertion is better than ETT intubation to maintain stable intraocular pressure. A random-effect model was employed to estimate the pooled standard mean differences due to severe heterogeneity (*I*^2^ 79.45,  *p* ≤ 0.001).

**Conclusion:**

The available information suggests that the LMA provides lesser intraocular pressure response in comparison with the conventional tracheal tube.

## 1. Background

Laryngeal mask airway (LMA) and endotracheal intubation are among the most important artificial airway devices used during delivery of general anesthesia [1]. Traditionally, laryngoscope and endotracheal tube (ETT) insertion has been the mainstay in providing adequate airway management [2]. Ophthalmic surgery traditionally requires general anesthesia with tracheal intubation that may have deleterious effects on intraocular pressure (IOP) [3]. LMA has been found to be superior to tracheal intubation in terms of maintaining stable IOP [4].

Laryngoscope and tracheal intubation cause an increase in sympathetic and sympathoadrenal activity in response to oropharyngeal, laryngeal, and tracheal stimulation. Both tracheal intubation and laryngeal mask airway insertion are noxious stimuli, which manifest as an increase in IOP lasting for approximately 5 min [5, 6]. Many studies in adults as well as in children have shown that the introduction of LMA causes less hemodynamic response and therefore lesser increase in IOP compared to insertion of a tracheal tube (TT) [7–9].

Normal IOP is 10–22 mmHg (mean 15 mmHg). There can be a 1–2 mmHg change during ventricular contraction, and a 1–6 mmHg change depending on the body positioning of the patient. IOP is also affected by blood pressure, respiration, coughing, the Valsalva maneuver, blinking, pressure from masks, and endotracheal intubation. Most anesthetic drugs affect IOP dose dependently. Barbiturates, muscle relaxants, opioids, sedatives, etomidate, and propofol can lower the normal IOP [10].

Control of intraocular pressure (IOP) during ophthalmic surgery is clinically important as elevation of IOP can cause the transient loss of vision or acute glaucoma [6]. Tracheal intubation may be associated with an acute increase in IOP, secondary to stress of the laryngoscope and passage of the endotracheal tube through the glottic aperture. Insertion of the LMA does not require a laryngoscope, but the introduction of the device and inflation of its cuff stimulate and exert pressure on the anterior pharyngeal wall [11, 12].

The increased intraocular pressure, blood pressure, and heart rate occurring due to reflex sympathetic discharge from response of laryngotracheal stimulation may have little consequences in healthy individuals, but may be more severe or even dangerous in patients with hypertension, myocardial insufficiency, and cardiovascular disease [3, 4]. In addition, the sudden rise in blood pressure can lead to left ventricular failure, cerebral hemorrhage, and myocardial ischaemia [5].

Many studies have shown the effect of different combinations of anesthetic drugs on reducing the side effects of tracheal intubation on patients' hemodynamic parameters and intraocular pressure [9, 10]. Despite of the combinations of various anesthetic agents used, there was a higher increase in hemodynamic changes in the ETT group than in the LMA group [11, 12]. However, a significant difference was not observed with propofol anesthesia [13]. Similarly, administering sevoflurane and remifentanil combination did not find significant difference in hemodynamic changes [14].

Many drugs and techniques have been used to attenuate the pressure responses following insertion of ETT, but no single technique has gained universal acceptance. Use of LMA in place of the ETT tube has been shown to have less hemodynamic response after insertion, as its insertion requires neither visualization of the cords nor penetration of the larynx which makes the placement less stimulating than the laryngoscope and ETT insertion. It may also provide less sympathetic responses and catecholamine releases [13, 14]. The aim of the present study was to perform a meta-analysis of all randomized controlled trial studies comparing effects of LMA and ETT on changes of IOP on all ophthalmic surgical patients.

## 2. Methods

### 2.1. Types of Participants

Participants were patients who underwent all kinds of ophthalmic surgery under general anesthesia.

### 2.2. Types of Intervention(s)/Phenomena of Interest

This review considered studies that evaluated the effects of tracheal intubation and laryngeal mask insertion on intraocular pressure after administering drugs which did not increase IOP and making sure that the patient is in deep level of general anesthesia before insertion of LMA and ETT. We excluded patients with DM, hypertension, gastroesophageal reflex, respiratory disease, and known eye diseases like glaucoma.

### 2.3. Types of Outcome

This review considered those studies that included the IOP as an outcome and had the SD and mean of IOP. We used IOP that was measured through a hand-held SchiÖtz tonometer and a Perkins hand-held applanation tonometer.

### 2.4. Types of Studies

The study included published and unpublished randomized controlled trials (RCTs). The inclusion criteria were all randomized control trial study designs with intervention LMA and comparator ETT.

### 2.5. Search Strategy

The search aim was to find both published and unpublished RCT studies. The search was restricted to studies published in the English language prior to March 4, 2019. A three-step search strategy was utilized in this review. An initial limited search of PubMed, Cochrane Database, CINAHL, Mednar, and Google Scholar was undertaken, followed by analysis of the text words contained in the title and abstract and of the index terms used to describe the article. A second search using all identified keywords and index terms was then undertaken across all included databases. Thirdly, the reference list of all identified reports and articles was searched for additional studies. We performed the literature search using the medical subject headings (MeSH) and text words related to laryngeal mask airway, endotracheal tube intubation, and intraocular pressure, ophthalmic surgical, and nonophthalmic surgical patients.

The following terms with MeSH (medical subject heading) Boolean operators and text words were used to search in PubMed:

((((Pediatric[tw]OR cataract[tw]eye surgery[tw]cataract[tw]vitrectomy[tw]OR ophthalmic surgery[tw] OR surgery patient[tw]OR nonophthalmic surgery[tw]OR orthopedic surgery[tw]spinal surgery[tw] OR pediatric surgery[tw]adult[tw]) OR (“Conversion to Open Surgery”[Mesh] OR “Orthognathic Surgery”[Mesh] OR “Vitreoretinal Surgery”[Mesh] OR “Corneal Surgery, Laser”[Mesh]“Conversion to Open Surgery”[Mesh] OR “Orthognathic Surgery”[Mesh] OR “Vitreoretinal Surgery”[Mesh] OR “Corneal Surgery, Laser”[Mesh] OR “Refractive Surgical Procedures”[Mesh])) AND ((laryngeal mask airways[tw] OR LMA[tw] OR Laryngeal mask[tw] OR Airway management[tw] OR Supraglottic airway devices[tw]) OR (“Laryngeal Masks”[Mesh] OR “Airway Obstruction”[Mesh] OR “Airway Management”[Mesh]))) AND ((endotracheal intubation[tw] OR ETT[tw] OR airway management[tw] OR Intratracheal intubation[tw]) OR (“Airway Management”[Mesh] OR “Airway Extubation”[Mesh] OR “Airway Resistance”[Mesh] OR “Airway Obstruction”[Mesh] “Intubation”[Mesh] OR “Intubation, Intratracheal”[Mesh]))) AND ((Intraocular pressure [tw] OR IOP [tw]) OR (“Intraocular Pressure”[Mesh] OR “Papilledema”[Mesh]))

### 2.6. Reporting

The results of this review were reported based on the Preferred Reporting Items for Systematic Review and Meta-Analysis statement (PRISMA) guideline [15]. The protocol was registered in the PROSPERO database.

### 2.7. Assessment of Methodological Quality

Quantitative papers selected for retrieval were assessed by two independent reviewers for assessing whether they are true RCT methods by using standard critical appraisal instruments for randomized controlled trials from the Joanna Briggs Institute Meta-Analysis of Statistics Assessment for Review Instrument (JBI-MAStARI, Joanna Briggs Institute, University of Adelaide, Australia). After the assessment of retrieved papers, for inclusion in the review, both reviewers agreed that a cutoff score of 7 out of 13 was used to determine the acceptable quality for inclusion.

### 2.8. Data Extraction

Quantitative data were extracted from papers included in the review using the standardized data extraction tool from JBI-MAStARI. The data extraction format included author, year of publication, sample size, mean, standard deviation, type of population, and surgical type. Two independent reviewers (MS and GA) extracted the data and cross-checked to ensure consistency. Discrepancies were solved by discussion and repeating the procedure. The reviewer contacted the corresponding author(s) for further information whenever pertinent data were missing from the included studies, but response was not obtained from the author.

### 2.9. Statistical Analysis

Data were extracted in Microsoft Excel and then exported to STATA version 14 for further analysis. The *I*^2^ statistics test was used to quantify heterogeneity among studies [16]. Laird's random-effect model was used to estimate the pooled standard mean difference of IOP after airway instrumentation. Effect sizes were expressed as weighted mean differences, and their 95% confidence intervals were calculated for analysis. Forest plots including mean, standard deviation and confidence intervals (CI), *p* value, effect size, and heterogeneity (*I*^2^) were constructed. *I*^2^ test statistics were used to investigate the heterogeneity across the included studies, and a *p* value less than 0.05 was used to declare significant heterogeneity. Subgroup analysis was done by the type of surgical procedure and the population type to minimize the random variations between the point estimates of the primary studies. The findings of which statistical pooling was not possible were presented in a narrative form.

## 3. Result

**Figure 1:**
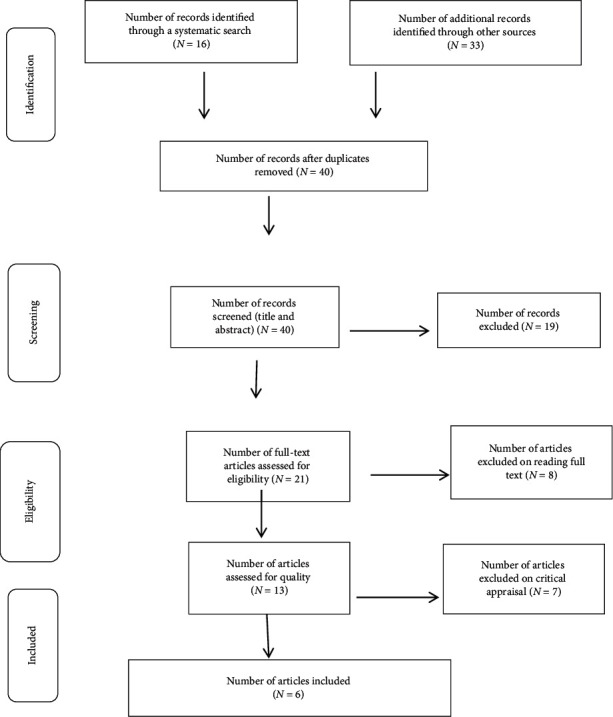
PRISMA flow diagram of IOP changes after insertion of LMA and ETT intubation.

This systematic review and meta-analysis included published and unpublished studies on the effect of endotracheal intubation and LMA insertion on intraocular pressure. The review found a total of 49 articles: 16 articles identified through a systematic search and 33 articles through other searches. From those, 9 duplicated records were removed, and 19 articles were excluded through screening of the title and abstracts due to irrelevance. After that, a total of 13 full-text papers were assessed for eligibility based on the inclusion and exclusion criteria. Finally, 6 studies were included for the final meta-analysis, and six articles were included for narrative reviews, while one which has not reported the figure was therefore excluded from both meta-analysis and narrative reviews (Figure 1).

### 3.1. Characteristics of the Included Studies

In the current systematic review and meta-analysis, 163 patients in the ETT group and 162 patients in the LMA group were included to estimate the pooled mean change of intraocular pressure. Eight studies were conducted to measure IOP rise in nonophthalmic procedures, and two ophthalmic procedures were involved. However, after instrumentation, two studies were not included in meta-analysis because of data incompleteness. The highest mean of IOP before the procedure 18 [17] and after the procedure 20.5 [18] was seen in the ETT group. On the other hand, the lowest mean changes before and after the procedure were found in the ETT group and the LMA group, respectively [19]. In this meta-analysis, two population categories were represented: two studies were from the pediatrics population and four studies were from the adult population (Tables 1 and 2).

### 3.2. Meta-Analysis before LMA Insertion and ETT Intubation

**Figure 2:**
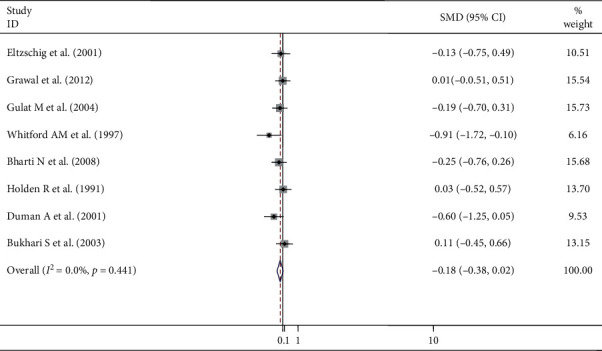
Meta-analysis of IOP changes before insertion of LMA and ETT intubation.

In this study, the pooled standard mean difference before ETT intubation and LMA insertion was −0.18 with a confidence interval of 0.38, 0.02. A fixed-effect model was employed to estimate the pooled standard mean difference of intraocular pressure. A heterogeneity of I-squared variation in SMD attributable to heterogeneity was 0.0% with heterogeneity chi-squared of 6.89 (d.f. = 7) (*p* = 0.441), showing there is no variation between the groups before instrumentation (Figure 2).

**Figure 3:**
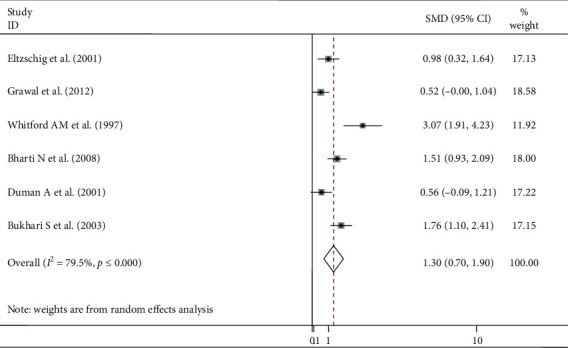
Meta-analysis of IOP after ETT intubation and LMA insertion.

**Figure 4:**
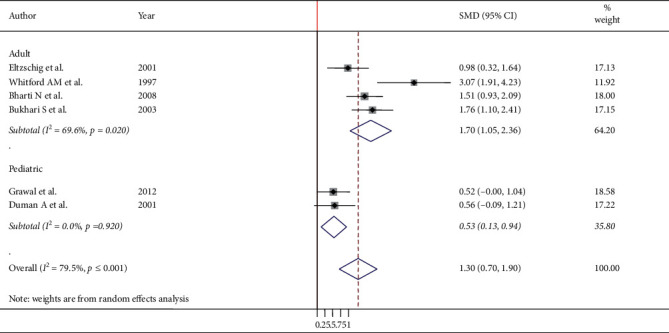
Subgroup analysis of IOP after ETT intubation and LMA insertion by the population type.

**Figure 5:**
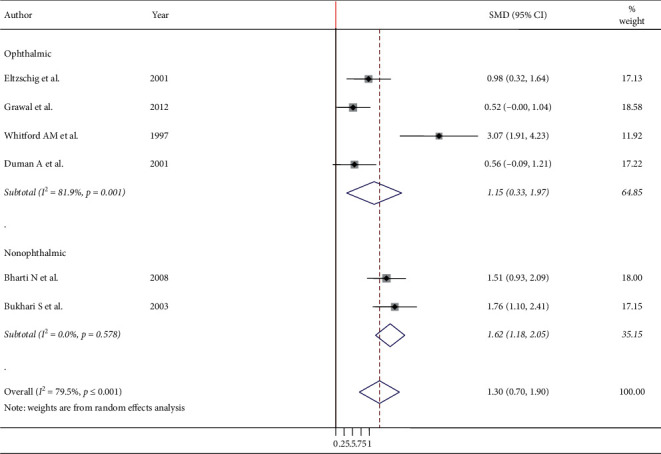
Subgroup analysis of IOP after ETT intubation and LMA insertion by the surgical type.

Six studies were used to examine the mean difference after endotracheal intubation and LMA insertion (Figure 3). The meta-analysis results showed that the overall pooled SMD of intraocular pressure of patients who underwent ETT intubation was higher by the mean difference of 1.30 (95% CI: 0.70, 1.90), showing that LMA insertion is better than ETT intubation to maintain stable intraocular pressure. A random-effect model was employed to estimate the pooled SMD due to severe heterogeneity (*I*^2^ 79.45, *p* ≤ 0.001) (Figure 3). Furthermore, subgroup analysis was done by surgical type and population type, and the result showed that adult population and ophthalmic surgical procedure had a significant heterogeneity with a determined value of *I*^2^ 69.6, *p* value = 0.020 (Figure 4), and *I*^2^ 81.9, *p* ≤ 0.001 (Figure 5), respectively. Subgroup analysis also showed that the pooled SMD in the ophthalmic and nonophthalmic procedure was 1.146 (95% CI, 0.325, 1.966) and 1.618 (95% CI, 1.185, 2.051), respectively, and the pooled SMD in adult and pediatric populations was 1.702 (95% CI, 1.048, 2.356) and 0.535 (95% CI, 0.129, 0.941), respectively. This showed that higher pooled SMD of intraocular pressure between ETT and LMA was found in the adult population.

### 3.3. Studies Included in the Narrative Review

Five studies were used as a narrative review of which a majority of studies revealed that both LMA and ETT were associated with significant intraocular pressor responses after airway instrumentation in both eyes; though, the mean maximum increase was significantly higher after tracheal intubation (Table 3).

## 4. Discussion

The stress response leading to increases in intraocular pressure during laryngoscope and tracheal intubation has been well documented. Such changes are likely to be harmful to the patients with hypertension and cardiovascular disease, glaucoma, penetrating eye injury, or an intracranial space-occupying lesion [20]. Various techniques have been tried to attenuate this response, but none has been completely successful. The laryngeal mask airway offers some advantages over conventional laryngoscope intubation with less pharyngolaryngeal stimulation [21] but with limitations [22].

This systematic review and meta-analysis found that the pooled standard mean difference between endotracheal tube intubation and LMA insertion was 1.30 (95% CI, 0.70, 1.90), showing that LMA insertion is better than ETT intubation to maintain stable intraocular pressure. Studies conducted to compare effects of laryngeal mask airway with tracheal tube on intraocular pressure for ophthalmic surgery in pediatric patients showed that endotracheal intubation causes higher mean change of intraocular pressure when compared to LMA insertion [23, 24]. However, another study revealed that there were no significant differences in intraocular pressure between LMA and ETT groups immediately after airway instrumentation except in the 5th min when intraocular pressure was 7.9 ± 2.3 mmHg in LMA and 9.4 ± 2.5 mmHg in the ETT group [14].

Subgroup analysis also showed that the pooled standard mean differences of IOP in the ophthalmic and nonophthalmic procedure showed that higher standard mean differences were found in the nonophthalmic procedure 1.618 (95% CI, 1.185, 2.051) when compared to the ophthalmic procedure 1.146 (95% CI, 0.325, 1.966). This also means that in both ophthalmic and nonophthalmic procedures, a higher mean change of intraocular pressure was found in endotracheal intubation as compared to LMA insertion. In addition, subgroup analysis of this systematic review and meta-analysis revealed that the pooled standard mean differences of IOP in adult and pediatric population showed that higher standard mean differences were found in adult populations 1.702 (95% CI, 1.048, 2.356) as compared to pediatric populations 0.535 (95% CI, 0.129, 0.941). Furthermore, most studies conducted to determine the effects of endotracheal intubation and laryngeal mask airway insertion indicated that laryngeal mask airway has a lesser intraocular pressor response [7, 17–19, 25, 26]. Another study done to measure the change in intraocular pressure on both right and left eyes showed that both groups were associated with significant intraocular pressor responses; nevertheless, the mean maximum increase was significantly higher after tracheal intubation [27]. However, there is no single study comparing the effects of endotracheal intubation and laryngeal mask airway insertion on intraocular pressor response.

## 5. Conclusion

The available information suggests that the LMA provides lesser intraocular pressure response in comparison with the conventional tracheal tube.

## Figures and Tables

**Table 1:** Description of the studies included in the meta-analysis before insertion of LMA and ETT intubation.

Author	Year	ETT group			LMA group			Surgery type	Population type
		Sample size 1	Mean 1	SD1	Sample_size 2	Mean 2	SD2		
Eltzschig et al.	2001	20	13.8	3.55	20	14.2	2.5	Ophthalmic	Adult
Gulati et al.	2004	30	13.1	4	30	13.9	4.3	Ophthalmic	Pediatrics
Grawal et al.	2012	29	13.12	3.63	30	13.12	3.95	Ophthalmic	Pediatrics
Whitford et al.	1997	13	12.54	1.73	13	16.06	3.02	Ophthalmic	Adult
Bharti et al.	2008	30	7.2	1.4	30	7.6	1.8	Nonophthalmic	Adult
Holden et al.	1991	26	18	4.1	26	17.9	3.8	Ophthalmic	Adult
Duman et al.	2001	20	11.5	2.72	18	13.08	2.51	Ophthalmic	Pediatrics
Bukhari et al.	2003	25	9.32	1.9	25	9.1	2.16	Nonophthalmic	Adult

**Table 2:** Description of the studies included in the meta-analysis after insertion of LMA and ETT intubation.

Author	Year	ETT group			LMA group			Surgery type	Population type
		Sample size 1	Mean 1	SD1	Sample size 2	Mean 2	SD2		
Eltzschig et al.	2001	20	15	4.83	20	10.81	3.61	Ophthalmic	Adult
Grawal et al.	2012	29	17.038	3.89	30	14.88	4.41	Ophthalmic	Pediatrics
Whitford et al.	1997	13	20.5	1.96	13	13.4	2.82	Ophthalmic	Adult
Bharti et al.	2008	30	16.8	5.3	30	10.4	2.8	Nonophthalmic	Adult
Duman et al.	2001	20	15.35	2.88	18	13.74	2.86	Ophthalmic	Pediatrics
Bukhari et al.	2003	25	16.62	3.15	25	11.52	2.63	Nonophthalmic	Adult

**Table 3:** Description of studies included in the narrative review.

No	Authors	Year	Study design	Surgical procedure	Outcome assessed
1	Gulati et al.	2004	RCT	Ophthalmic pediatrics	There was no significant change in mean intraocular pressure after insertion of the LMA. However, in the endotracheal tube group, the mean intraocular pressure significantly increased from a baseline of 13.1 ± 4.0 mmHg to 19.9 ± 7.3 mmHg.

2	Myint et al.	1995	RCT	Ophthalmic adult	Intraocular pressures were lower than baseline in both groups throughout anesthesia. But one min after removal of the device, mean intraocular pressure in the tracheal tube group was 16.0 mmHg and was significantly higher than the laryngeal mask group (10.9) (*p* < 0.01).

3	Ziyaeifard et al.	2012	RCT	Ophthalmic adult	There were no significant differences in IOP between LMA and ETT groups immediately after airway instrumentation except in 5th min when IOP was 7.9 ± 2.3 mmHg in LMA and 9.4 ± 2.5 mmHg in the ETT group; (*p* = 0.030).

4	Holder et al.	1991	RCT	Ophthalmic adult	Mean IOP before airway instrumentation in LMA and ETT groups was 17.9 ± 3.8 and 18.±4.1. However, after airway instrumentation, mean changes in LMA was 1.8 ± 21 and 6.8 ± 5.5 in the LMA group.

5	Ghai et al.	2001	RCT	Ophthalmic adult	IOP was measured in both right and left eyes. Both groups were associated with significant intraocular pressor responses after airway instrumentation in both eyes; however, the mean maximum increase was significantly higher after tracheal intubation.

## Data Availability

Extracted data are incorporated into the manuscript as Tables 1 and 2.
